# Impact of the COVID-19 Epidemic on Lifestyle Behaviors and Their Association With Subjective Well-Being Among the General Population in Mainland China: Cross-Sectional Study

**DOI:** 10.2196/21176

**Published:** 2020-08-25

**Authors:** Zhao Hu, Xuhui Lin, Atipatsa Chiwanda Kaminga, Huilan Xu

**Affiliations:** 1 Department of Social Medicine and Health Management Xiangya School of Public Health Central South University Changsha China; 2 Department of Mathematics and Statistics Mzuzu University Luwinga, Mzuzu Malawi; 3 Department of Epidemiology and Health Statistics Xiangya School of Public Health Central South University Changsha China

**Keywords:** COVID-19, coronavirus disease, subjective well-being, lifestyle behaviors

## Abstract

**Background:**

The world is experiencing an unprecedented challenge due to the coronavirus disease (COVID-19) pandemic. However, it is unclear whether people’s lifestyles will change as a result.

**Objective:**

The aim of this study is to explore perceived lifestyle changes after the outbreak of COVID-19 and their association with subjective well-being (SWB) among the general population in Mainland China.

**Methods:**

An online survey was conducted in May 2020. Lifestyle behaviors including leisure-time physical exercise, leisure-time screen time, and dietary intake were self-reported. SWB was measured using the General Wellbeing Schedule (GWS). Other covariates including sociodemographic factors, self-rated physical health, perceived social support, and loneliness were also assessed by a structured questionnaire. A multivariate ordinal regression method was used to analyze the association between SWB and lifestyle behaviors as well as perceived lifestyle changes.

**Results:**

A total of 1033 participants aged between 18 and 60 years were included in this study. The mean GWS score was 71.7 points. About 70% of the respondents reported spending more time looking at screens, whereas about 30% reported an increased frequency of vegetable and fruit intake after the outbreak of COVID-19. Inactive physical exercise (odds ratio [OR] 1.16, 95% CI 1.02-1.48), infrequent vegetable intake (OR 1.45, 95% CI 1.10-1.90), infrequent fruit intake (OR 1.31, 95% CI 1.01-1.70), and often skipping breakfast (OR 1.43, 95% CI 1.08-1.91) were associated with lower SWB after adjusting for sociodemographic factors, self-rated physical health, perceived social support, and loneliness. Moreover, participants who perceived a decrease in the frequency of vegetable, fruit, and breakfast intake were more likely to report lower SWB.

**Conclusions:**

The COVID-19 pandemic may have positive and negative impacts on different aspects of lifestyle behaviors. Both unhealthy lifestyle behaviors and negative lifestyle changes were associated with lower SWB. These findings provide scientific evidence that can inform lifestyle guidelines and public mental health interventions during the COVID-19 outbreak.

## Introduction

The ongoing coronavirus disease (COVID-19) outbreak has led to an unprecedented public health crisis worldwide [1]. COVID-19, similar to severe acute respiratory syndrome (SARS), is a beta-coronavirus that can be spread to humans through intermediate hosts such as bats [2]. The COVID-19 outbreak was first revealed in late December 2019 in Wuhan, Hubei Province, China. On January 30, 2020, the World Health Organization (WHO) declared the COVID-19 outbreak a public health emergency of international concern [3]. In the following months, COVID-19 spread rapidly in China and worldwide.

At the same time, the global population is experiencing life-altering challenges due to the COVID-19 pandemic [4]. Given the lack of effective treatment for COVID-19, nonpharmacological interventions (NPIs) are required to decrease its transmission [5]. In essence, NPIs are intended to modify disease-related lifestyle behaviors such that these should no longer contribute to the spread of the disease. However, lifestyle choices or behavioral change may lead to unforeseen detrimental or protective consequences for mental health outcomes [6-10]. For example, some NPIs, such as personal restrictions, mass confinement, and compulsory home isolation, may be associated with worse psychological conditions [8], whereas some NPIs, such as good ventilation in the workplace and wearing a face mask, were found to be protective of mental health during the COVID-19 pandemic [9,10]. Furthermore, evidence from recent studies suggests that current pandemic-related coping strategies may have an adverse impact on mental health, such as decreased well-being and increased posttraumatic stress disorders (PTSD), depression and anxiety symptoms, insomnia, and anger [11-13]. In addition, fear of the disease and social isolation may lead to stress reactions that could develop into other psychological disorders [14].

Unhealthy lifestyle habits such as poor diet, lack of physical activity, smoking, and alcohol use are not only major contributors to the global burden of disease [15], but are also positively associated with worse mental health outcomes [16]. Recently, lifestyle guidelines have emphasized maintaining a healthy nutritional status and engaging in physical exercise at home during the COVID-19 outbreak [17,18]. Many other studies have suggested that the focus should be on addressing the mental health aspect when implementing public disease control and prevention interventions [19,20]. However, observational studies on the characteristics of behavior patterns such as dietary intake, physical exercise, and screen time among the general population after the outbreak of COVID-19 are lacking [21]. Similarly, data about perceived lifestyle changes among the general population after the outbreak of COVID-19 are also lacking. Moreover, the associations between mental health outcomes and lifestyle behaviors as well as lifestyle changes during the COVID-19 pandemic represent a research gap.

Therefore, this study aimed to explore the perceived lifestyle changes after the outbreak of COVID-19, and their association with subjective well-being among the general population in Mainland China. It is hoped that, from the public health and preventative care perspectives, this study can provide valuable information to inform public health policies or interventions aimed at maintaining good mental health and a healthy lifestyle during the COVID-19 pandemic.

## Methods

### Overview

This study was conducted from May 10 to May 15, 2020, through an online survey using an internet platform [22]. This platform has more than 2.6 million members distributed across more than 30 provinces in China, of which 52.00% are female, 29.34% are aged 26 to 30 years, and 39.20% are general staff. All participants signed an online informed consent form before filling out this questionnaire. The target population comprised all Chinese people in Mainland China aged between 18 and 60 years. A computer-assisted simple random sampling method was used to select eligible participants from this internet platform. Adolescents were not interviewed because it would be difficult to obtain parental consent over the internet, and older adults (≥60 years old) were not investigated as recall bias may be strong in this group. Participants were also excluded if they refused to participate in this survey, did not reply, or could not complete the online survey independently.

### Data Collection

#### Sociodemographic Information

A self-administrated structured questionnaire was used to collect the participants’ sociodemographic information including age, gender (male or female), residential location (urban or rural area), marital status (married or unmarried), education (high school and below, college or university degree, master’s degree and above) and personal monthly income. The unmarried group included those who were divorced, single, and widowed.

#### Lifestyle Behaviors Assessment

Lifestyle behaviors in the 4 months immediately before and the 4 months immediately after the COVID-19 outbreak were assessed. In addition, the perceived lifestyle changes after the COVID-19 outbreak were assessed in relation to lifestyle behaviors in the 4 months before the COVID-19 outbreak. The specific lifestyle behaviors that were assessed included the following: leisure-time physical exercise, leisure-time screen time, and dietary intake. To measure leisure-time physical exercise, four items from the long version of the International Physical Activity Questionnaire (IPAQ), a valid and reliable questionnaire assessing physical activity [23], were used. Of the four items, two assess the frequency of moderate or vigorous physical exercise (such as running and dancing) per week and the other two items assess the duration of physical exercise every time. Participants were defined as active if they reported a total moderate-vigorous physical exercise time of more than 150 minutes/week [24]. The Cronbach α of these items in this study was 0.685. Leisure-time screen time was viewed as a major indicator of sedentary behavior, and was calculated by summing up the screen time related to watching TV/videos, internet use (such as news and Douban) through a smartphone or the internet, playing computer or smartphone games, studying online, and social platform use (such as QQ and Wechat). Leisure-time screen time was further classified as short (<2 hours/day) and long (≥2 hours/day). Three items from the simplified food frequency questionnaire (FFQ-25) [25] were used to assess the weekly frequency of consuming vegetables (including light- and deep-colored vegetables) and fruits (such as apples and pears). Thus, participants were dichotomized as frequently (≥5 times/week) and infrequently (<5 times/week) consuming fruits and vegetables. The Cronbach α of these items in this study was 0.725. The question “How many days do you skip breakfast weekly after the outbreak of COVID-19?” was used to assess the frequency of skipping breakfast and this dichotomized participants as seldom (<3 days/week) and often (≥3 days/week). Perceived lifestyle changes compared to lifestyle behaviors before the outbreak of COVID-19 were classified into three categories as follows: increased or much increased, same as before, and decreased or much decreased.

#### Covariates

Other covariates assessed were self-rated physical health, perceived social support (PSS), and loneliness during the COVID-19 epidemic. Self-rated physical health was assessed using a 5-item Likert scale. For example, one of the questions asked, “How do you feel about your physical condition?” Participants rated their physical condition as good, fair, or poor. PSS was measured using the Chinese version of the Perceived Social Support Scale (PSSS). The PSSS was developed by Zimet in 1988 [26] and was used to measure PSS from three sources: family, friends, and significant others. This scale contain 12 items, scored on a 7-point rating scale from 1 (very strongly disagree) to 7 (very strongly agree). The total score ranges from 12 to 84, and PSS is divided into low (a score from 12 to 36), intermediate (a score from 37 to 60), and high (a score from 61 to 84). The Cronbach α of this scale in this study was 0.881. Loneliness was measured using the short from of the University of California, Los Angeles (UCLA) Loneliness Scale (ULS-8) [27], which was adapted by Hays and DiMatteo in 1987 on the basis of UCLA-20 [28]. This scale contains 8 items and the total score ranges from 8 to 32, with higher scores indicating higher levels of loneliness. The loneliness of participants was classified into three levels (low, intermediate, and high) according to the tertile of total score of ULS-8. The Cronbach α of this scale in this study was 0.818.

#### Subjective Well-Being Assessment

Subjective well-being (SWB), a main indicator of mental health, is a broad category of phenomena that includes people’s emotional response, domain satisfaction, and global judgments of life satisfaction [29]. SWB was measured using the Chinese adapted version of the General Wellbeing Schedule (GWS) [30], which was first developed by the US National Center for Health Statistics in 1977. It contains the following 6 dimensions: satisfaction and interest in life; health concerns; energy; depression or pleasant mood; control of emotions and behavior; and relaxation and tension. The total scores for this instrument are yielded by summing up the items’ scores, with higher scores indicating higher levels of SWB. The SWB of participants was classified into three levels (high, intermediate, and low) according to the tertile of total scores on the GWS. The GWS has high reliability and validity in the Chinese population, with a Cronbach of 0.91 and 0.95 for men and women, respectively [30]. The Cronbach α of this scale in this study was 0.812.

### Statistical Analysis

Categorical variables were summarized as counts and percentages, while continuous variables were summarized as mean and standard deviation. The statistical difference in the distribution of the GWS scores across different sociodemographic characteristics and lifestyle behaviors was assessed using the Student *t* test or the one-way analysis of variance (ANOVA). The statistical difference in the distribution of perceived lifestyle changes across different groups was assessed using the chi-square test. Multivariate ordinal regression models were used to explore the association between SWB (1=low; 2=intermediate; 3=high) and lifestyle behaviors as well as perceived lifestyle changes. The strength of association was reported as odds ratio (OR) and 95% CI. An unadjusted relationship between SWB and each lifestyle behavior as well as its change was examined in Model 1 (crude model), whereas an adjusted relationship between SWB and each lifestyle behavior as well as its change was examined in Model 2, adjusted for the variables age, gender, education, marital status, residential location, and personal monthly income. Finally, a relationship between SWB and each lifestyle behavior as well as its change was examined in Model 3, which adjusted for covariates including self-rated physical health, perceived social support, loneliness, and all the covariates entered in Model 2. The statistical analyses were conducted in SPSS (Version 22.0; IBM Corp). All statistical tests were two-tailed and a *P*<.05 was considered statistically significant.

## Results

A total of 1600 registrants were invited to take part in this online survey and a total of 1081 subjects consented to take part in the study, providing a response rate of 67.6%. The main reasons for nonresponse were declining to participate and no reply. After excluding 48 participants due to an incomplete questionnaire or missing data, a total of 1033 participants were included in this study. Among them, 637 participants were aged between 18 and 30 years (61.7%) and 498 were female (48.2%). Further, a very small proportion of the participants (n=95, 9.2%) had attended high school and lower levels of education. In addition, 730 subjects (70.7%) were living in urban areas, 846 participants (81.9%) considered their physical health status as good, and 675 participants (65.3%) felt that they had been receiving high social support. Regarding lifestyle, 58.8% (n=607) of the participants had leisure-time physical exercise of more than 150 min/week, about 86.8% (n=897) had screen time of 2 hours/day or more, and 67.9% (n=701) ate vegetables 5 times/week or more, whereas 41.8% (n=432) of the subjects ate fruits 5 times/week or more. Characteristics of participants are summarized in Table 1.

The mean GWS score in this sample was 71.7 points (SD 12.5). In addition, the distribution of GWS scores was not significantly different across the categories of gender, education, and leisure-time screen time (*P*>.05). Table 1 shows the details.

**Table 1:** Distribution of sample characteristics and General Wellbeing Schedule scores.

Characteristics		Participants, n (%)	General Wellbeing Schedule score, mean (SD)	*P* value^a^
**Age (years)**				
	18-30	637 (61.7)	70.9 (12.1)	.03
	31-40	281 (27.2)	72.3 (12.6)	N/A^b^
	≥41	115 (11.1)	74.1 (14.3)	N/A
**Gender**				
	Male	535 (51.8)	71.8 (12.2)	.68
	Female	498 (48.2)	71.5 (12.9)	N/A
**Residential location**				
	Urban	730 (70.7)	72.6 (12.5)	<.001
	Rural	303 (29.3)	69.5 (12.4)	N/A
**Education**				
	High school and below	95 (9.2)	69.5 (12.2)	.06
	College or university degree	858 (83.1)	71.7 (12.5)	N/A
	Master’s degree and above	80 (7.7)	74.1 (12.5)	N/A
**Marital status**				
	Married	485 (47.0)	73.2 (12.4)	<.001
	Unmarried	548 (53.0)	69.9 (12.5)	N/A
**Personal monthly income (¥)**				
	<3000	283 (27.4)	69.2 (12.4)	<.001
	3000-7999	471 (45.6)	71.2 (12.4)	N/A
	≥8000	279 (27.0)	74.9 (12.3)	N/A
**Self-rated physical health**				
	Good	846 (81.9)	73.6 (11.8)	<.001
	Fair	177 (17.1)	63.4 (12.0)	N/A
	Poor	10 (1.0)	54.7 (11.6)	N/A
**Perceived social support**				
	High	675 (65.3)	75.7 (12.5)	<.001
	Intermediate	340 (32.9)	64.8 (11.3)	N/A
	Low	18 (1.8)	52.3 (16.3)	N/A
**Loneliness**				
	High	340 (32.9)	81.0 (9.3)	<.001
	Intermediate	387 (37.5)	71.1 (10.1)	N/A
	Low	306 (29.6)	62.0 (10.7)	N/A
**Leisure-time physical exercise**				
	Active	607 (58.8)	72.9 (12.4)	<.001
	Inactive	426 (41.2)	69.9 (12.6)	N/A
**Leisure-time screen time**				
	Short	136 (13.2)	72.1 (14.0)	.64
	Long	897 (86.8)	71.6 (12.3)	N/A
**Frequency of vegetable intake**				
	Infrequently	332 (32.1)	69.2 (12.1)	<.001
	Frequently	701 (67.9)	72.8 (12.6)	N/A
**Frequency of fruit intake**				
	Infrequently	601 (58.2)	69.8 (12.0)	<.001
	Frequently	432 (41.8)	74.2 (12.8)	N/A
**Frequency of skipping breakfast**				
	Seldom	721 (69.8)	73.4 (12.5)	<.001
	Often	312 (30.2)	67.7 (11.7)	N/A

^a^*P* values were obtained according to the Student *t* test or one-way analysis of variance.

^b^N/A: not applicable.

Furthermore, about 17.0% of the respondents stated that they spent more time doing physical exercise, and about 2/3 of the respondents reported that they spent more time looking at screens. Additionally, a small proportion of participants decreased the frequency of their intake of vegetables, fruits, and breakfast. In addition, changes in the frequency of fruit and breakfast intake were associated with participants’ residential locations. There were no statistical differences in perceived lifestyle changes associated with gender. These results are shown in Table 2.

**Table 2:** Perceived lifestyle changes by gender and place of residence.

Lifestyle habits		Overall, %	Gender			Place of residence		
			Male, %	Female, %	*P* value^a^	Urban, %	Rural, %	*P* value^a^
**Time spent exercising**								
	Increased or much increased	17.8	18.5	17.1	.12	18.6	19.5	.36
	Same as before	63.3	65.1	61.4	N/A^b^	62.5	65.3	N/A
	Decreased or much decreased	18.9	16.4	21.5	N/A	18.9	15.2	N/A
**Time spent looking at screens**								
	Increased or much increased	68.3	65.2	71.5	.09	70.0	64.0	.12
	Same as before	23.9	25.8	21.9	N/A	23.0	26.1	N/A
	Decreased or much decreased	7.8	9.0	6.6	N/A	7.0	9.9	N/A
**Frequency of vegetable intake**								
	Increased or much increased	28.8	28.4	29.1	.32	29.2	27.7	.89
	Same as before	55.8	54.6	57.2	N/A	55.5	56.8	N/A
	Decreased or much decreased	15.4	17.0	13.7	N/A	15.3	15.5	N/A
**Frequency of fruit intake**								
	Increased or much increased	35.3	36.7	33.9	.21	37.1	31.0	.004
	Same as before	46.0	46.7	45.2	N/A	46.7	44.2	N/A
	Decreased or much decreased	18.7	16.6	20.9	N/A	16.2	24.8	N/A
**Frequency of skipping breakfast**								
	Increased or much increased	23.1	20.2	26.1	.06	23.4	22.1	.02
	Same as before	56.9	58.1	55.6	N/A	58.8	52.5	N/A
	Decreased or much decreased	20.0	21.7	18.3	N/A	17.8	25.4	N/A

^a^*P* value was obtained by chi-square test.

^b^N/A: not applicable.

The association between lifestyle behaviors during the COVID-19 pandemic and subjective well-being among the general population in Mainland China is summarized in Table 3. The multivariate ordinal regression model showed that participants with inadequate leisure-time physical exercise (OR 1.16, 95% CI 1.02-1.48), infrequent vegetable intake (OR 1.45, 95% CI 1.10-1.90), infrequent fruit intake (OR 1.31, 95% CI 1.01-1.70), as well as those who often skipped breakfast (OR 1.43, 95% CI 1.08-1.91) were associated with a higher risk of lower subjective well-being after adjusting for age, gender, education, marital status, residential location, personal monthly income, self-rated physical health, perceived social support, and loneliness.

The association between perceived lifestyle changes, before and after the outbreak of COVID-19, and subjective well-being is summarized in Table 4. An ordinal regression model indicated that participants with decreased frequency of vegetable and fruit intake, and increased frequency of skipping breakfast were more likely to report lower subjective well-being after adjusting for sociodemographic factors and other covariates.

**Table 3:** The association between lifestyle behaviors and subjective well-being.

Lifestyle behaviors		Model 1, OR^a^ (95% CI)	Model 2^b^, OR (95% CI)	Model 3^c^, OR (95% CI)
**Leisure-time physical exercise**				
	Active	1.00	1.00	1.00
	Inactive	1.51 (1.20-1.89)	1.42 (1.12-1.79)	1.16 (1.02-1.48)
**Leisure-time screen time**				
	Short	1.00	1.00	1.00
	Long	1.12 (0.81-1.57)	1.09 (0.78-1.53)	1.37 (0.94-1.99)
**Frequency of vegetable intake**				
	Frequently	1.00	1.00	1.00
	Infrequently	1.82 (1.41-2.30)	1.81 (1.41-2.31)	1.45 (1.10-1.90)
**Frequency of fruit intake**				
	Frequently	1.00	1.00	1.00
	Infrequently	1.88 (1.49-2.37)	1.70 (1.34-2.14)	1.31 (1.01-1.70)
**Frequency of skipping breakfast**				
	Seldom	1.00	1.00	1.00
	Often	2.28 (1.78-2.92)	2.12 (1.64-2.73)	1.43 (1.08-1.91)

^a^OR: odds ratio.

^b^Adjusted for age, gender, marital status, residential location, education, and personal monthly income.

^c^Adjusted for covariates in Model 2 and plus self-rated physical health, perceived social support, and loneliness.

**Table 4:** The association between perceived lifestyle changes and subjective well-being.

Perceived lifestyle changes	Model 1, OR^a^ (95% CI)	Model 2^b^, OR (95% CI)	Model 3^c^, OR (95% CI)
Decreased time spent exercising	1.15 (0.85-1.54)	1.14 (0.86-1.56)	1.11 (0.80-1.54)
Increased time spent looking at screens	1.33 (1.05-1.69)	1.32 (0.98-1.60)	1.03 (0.80-1.34)
Decreased frequency of vegetable intake	1.84 (1.34-2.53)	1.78 (1.30-2.44)	1.73 (1.21-2.46)
Decreased frequency of fruit intake	1.56 (1.16-2.08)	1.48 (1.10-1.98)	1.41 (1.02-1.96)
Increased frequency of skipping breakfast	2.05 (1.45-2.88)	1.83 (1.29-2.59)	1.49 (1.01-2.18)

^a^OR: odds ratio.

^b^Adjusted for age, gender, marital status, residential location, education, and personal monthly income.

^c^Adjusted for covariates in Model 2 and plus self-rated physical health, perceived social support, and loneliness.

## Discussion

Previous studies have predominantly focused on the psychological impact of the COVID-19 epidemic, rather than lifestyle issues. For the first time, some perceived lifestyle changes after the outbreak of COVID-19 have been assessed, and the impact of such changes on mental health was also explored among the general population in Mainland China. Noticeably, NPIs have modified some lifestyle behaviors positively and others negatively. Both unhealthy lifestyle behaviors and negative lifestyle changes were associated with lower SWB. Although about half of the participants reported no lifestyle changes, the percentages of reported favorable lifestyle changes were larger than the percentages of reported unfavorable lifestyle changes, especially in relation to the frequency of vegetable and fruit intake. However, the situation was the opposite when considering leisure-time physical exercise and screen time. Thus, it is possible that the sudden occurrence of COVID-19 made people reconsider their healthy lifestyle habits. In addition, the social or home isolation policy made people avoid public places and increase their indoor time, which may have increased their use of electronic media at home. Cognitive behavior therapy enables activity scheduling during home isolation and improves mental health [31].

Accordingly, unhealthy lifestyle behaviors were recorded among the Chinese population after the outbreak of COVID-19. For example, about 40% of the participants had inactive leisure-time physical exercise and about 90% had longer screen time. In addition, vegetable and fruit intake was less than 5 times/week for about 30% and 60% of the participants, respectively. However, it is not known whether such lifestyle patterns would persist during the COVID-19 pandemic or after the COVID-19 pandemic. Therefore, further studies to assess the lasting effects of the COVID-19 pandemic on lifestyle behaviors are warranted.

The findings of this study have added to the existing evidence that physical exercise is associated with mental well-being [32,33]. For example, a larger cross-sectional study indicated that sports and vigorous recreational activities were positively associated with emotional well-being even after adjusting for sex, social class, and health status [34]. In addition, active physical exercises were associated with reduced risk of mental health conditions such as depression and anxiety [35]. Similarly, during the SARS epidemic, increased exercise time was associated with decreased perceived stress and incidence of PTSD in the general population of Hong Kong [36]. However, increased exercise time did not significantly predict subjective well-being. A possible explanation could be that SWB may be associated with the intensity of physical exercise. Many studies demonstrated that vigorous physical exercises were positively associated with SWB, while moderate physical exercises were either not associated or negatively associated with SWB [37,38]. Another reason is that the environment for physical exercise may have a great impact on mental well-being [39]. Therefore, further studies about intensity of physical exercises under different environments are needed to fully elucidate the effects on wellbeing.

The finding that a higher frequency of vegetable and fruit intake was positively associated with subjective well-being is consistent with previous studies [40-42]. There is an urgent need to disseminate this health information to the public during the COVID-19 pandemic via the internet and health collaborators [43,44]. For example, Stranges et al [45] reported that the odds for low mental well-being were increased in those with decreasing fruit and vegetable intake. Furthermore, a systematic review of 61 studies indicated that higher total intake of fruits and vegetables may promote higher levels of optimism and reduce psychological distress, thereby having a positive influence on mental health [42]. Even though the total quantities of vegetable and fruit intake were not obtained in this study, we assumed that a higher frequency of intake may lead to higher total volume intake; thus, the former and the latter may have the same influence. Nevertheless, further studies are warranted to understand the association between mental health and quantities of vegetables and fruits consumed. The finding about the relationship between eating habits, like skipping breakfast, and well-being added to the existing evidence that skipping breakfast is associated with poorer physical and mental health outcomes [41,46,47]. For instance, breakfast skippers had significantly worse health-related quality of life both physically and mentally in a Taiwanese national representative sample [48]. In addition, a large cohort study indicated that an eating pattern characterized by skipping or delaying breakfast was associated with mood disorders among Australian adults [49]. However, the relationship between mental health and the manner in which breakfast is eaten needs further investigation.

In summary, this original preliminary study examined some positive and negative lifestyle changes due to the influence of the COVID-19 pandemic. It has revealed the pressing need to provide individuals, communities, and health agencies with information to help maintain healthy lifestyles to some degree while in isolation. Moreover, this study has contributed to the scant literature on the association between lifestyle behaviors and mental health during the COVID-19 pandemic. However, this study has several limitations. First, this study had a cross-sectional design and was conducted during the COVID-19 pandemic. Thus, causality relationships could not be inferred, and it is not clear whether the association between subjective well-being and lifestyle behaviors as well as their changes will last medium- and long-term. Second, precise information about lifestyle behaviors was difficult to collect before the start of the COVID-19 pandemic. Therefore, only perceived lifestyle changes were measured. Moreover, lifestyle behaviors and perceived changes were self-reported, thus these measurements may be susceptible to recall bias. Third, this study population had a high proportion of subjects aged 18 to 40 years (approximately 90%) and a high proportion of participants had a higher education level. Whether the observed changes and associations are present in a more representative study population requires further exploration. Therefore, longitudinal studies with representative samples should be conducted during the COVID-19 pandemic to better understand the lasting effects of this pandemic on lifestyle behaviors and their changes.

## Abbreviations

COVID-19: coronavirus disease
FFQ-25: Food Frequency Questionnaire
GWS: General Wellbeing Schedule
NPI: nonpharmacological intervention
OR: odds ratio
PSS: perceived social support
PSSS: Perceived Social Support Scale
PTSD: posttraumatic stress disorder
SARS: severe acute respiratory syndrome
SWB: subjective well-being
ULS-8: University of California, Los Angeles Loneliness Scale
WHO: World Health Organization
